# *Escherichia coli* Strains Responsible for Cystitis in Female Pediatric Patients with Normal and Abnormal Urinary Tracts Have Different Virulence Profiles

**DOI:** 10.3390/pathogens11020231

**Published:** 2022-02-10

**Authors:** Marta de Oliveira Domingos, Silvio Marciano da Silva Junior, Wagner Milanello, Shirley Sizue Nakamura Nakano, Marcia Regina Franzolin, Luis Fernando dos Santos, Kamila Oliveira Nunes, Vaniky Duarte Marques, Waldir P. Elias, Herbert Guimarães de Sousa Silva, Bruna De Lucca Caetano, Roxane Maria Fontes Piazza

**Affiliations:** 1 Laboratório de Bacteriologia, Instituto Butantan, Avenida Vital Brasil, 1500, São Paulo 05503-900, SP, Brazil; silviomsj@yahoo.com.br (S.M.d.S.J.); marcia.franzolin@butantan.gov.br (M.R.F.); kamila.nunes1@gmail.com (K.O.N.); nikymd@hotmail.com (V.D.M.); waldir.elias@butantan.gov.br (W.P.E.); herbert.silva@esib.butantan.gov.br (H.G.d.S.S.); bruna3caetano@gmail.com (B.D.L.C.); roxane.piazza@butantan.gov.br (R.M.F.P.); 2 Hospital Infantil Darcy Vargas, Rua Seráfico de Assis Carvalho, 34, São Paulo 05614-040, SP, Brazil; shirleynakano@yahoo.com.br; 3 Centro de Bacteriologia, Núcleo de Doenças Entéricas, Instituto Adolfo Lutz, Avenida Dr. Arnaldo, 355, São Paulo 01246-000, SP, Brazil; luis.santos@ial.sp.gov.br

**Keywords:** cystitis1, *Escherichia coli* 2, UPEC 3, phylogeny 4, biofilm 5, normal and abnormal urinary tract 6

## Abstract

The role of uropathogenic *Escherichia coli* (UPEC) in colonization and infection of female patients with anatomical and functional abnormalities of the urinary system is elusive. In this study, the phenotype, genotype and the phylogeny of UPEC strains isolated from the urine of pediatric female patients with cystitis of normal and abnormal urinary tract were determined. Multiplex PCR results demonstrated that 86% of the strains isolated from female patients with normal urinary tract (NUT), belonged to the phylo-groups B2 and D. Their prevalence decreased to 23% in strains isolated from patients with abnormal urinary tract (AUT). More of the isolates from AUT patients produced a biofilm on polystyrene and polyvinyl chloride (PVC), adhered to epithelial cells, and encoded *pap* and *sfa* genes than strains isolated from female patients with NUT. In contrast, a higher number of hemolysin-producing strains with serogroups associated with UPEC were isolated from patients with NUT. In summary, the results suggest that cystitis in female patients with NUT is associated with ExPEC, whereas cystitis in female patients with AUT is associated with pathogenic intestinal *E. coli* strains that have acquired the ability to colonize the bladder.

## 1. Introduction

After respiratory infection, urinary tract infection (UTI) is the most common infection in children [1]. Although it is considered a benign infection in healthy adults, UTI may raise the risk of pyelonephritis, fetal mortality and renal complications in pediatric patients.

Several bacterial species can cause urinary tract infection, however, the main etiological agents responsible for community-acquired UTI worldwide, and a large portion of nosocomial UTIs, are uropathogenic *E. coli* [2,3].

UPEC strains have developed along evolution several fimbrial adhesins that help them to colonize the urinary tract [4]. The primary adherence fimbriae of UPEC are type 1, P and S, encoded by the operons *fimH*, *pap* and *sfa* respectively [5].

The type 1 fimbria is associated to the bacterial process of colonization and invasion of the host cells, while P and S fimbriae enhance the establishment of *E. coli* infection in the urinary tract and significantly lower the number of colony forming-units (CFU) necessary to cause UTI [6].

UPEC strains can also produce a wide variety of virulence factors such as hemolysin, Pic, Pet Sat and cytotoxic necrotizing type 1 (CNF-1) factor among others [7,8]. However, the prevalence of these factors differs from one strain to another, which may influence the prognosis of the disease [9].

Classical UPEC strains belong to the phylogenetic groups B2 and D, which includes most virulent extraintestinal *E. coli* strains [10,11,12]. They are also predominant in the following serogroups: O2, O6, O7, O8, O15, O16, O18, O21, O22, O25, O75 and O83 [13,14].

The most common type of UTI encountered in women is cystitis. Despite the fact that most cases of cystitis can easily be cured in patients with a normal urinary tract, it is not the same in the case of patients with anatomical malformation or with abnormal functionality of the urinary tract.

The anomalies of the urinary tract can be divided into nephropaties and uropathies [15]. Nephropaties are associated with disfunction and/or mal-formation of the kidneys whereas uropathies represent pathologies of the urinary tract which depending on the site of the anomaly, they can be pyeloureteral and ureterovesical, or they can relate to the vesicoureteral reflux and to the posterior urethral valve [15]. 

The most common mal formation of the urinary tract described in children are vesicoureteral reflux, obstructive megaureter, posterior urethral valve and megaystis.

The anomalies can also affect the bladder and expose its mucosa to the external environment. Some patients can also present rectal prolapse and anal incontinence [15].

In these patients, a clinical picture of cystitis can become more severe, since bacteria have better conditions to ascend to the kidneys and induce pyelonephritis. The use of catheters can also contribute to the severity of the infection, since they can become a platform for bacterial biofilm formation and infection [16].

Other factors that influence the development of UTI in women are age, hormone levels and sexual intercourse. For instance, in female pediatric patients the incidence of UTI is 0.4–1% in early age, increases to 0.9–1.4% between the ages 1 and 5 years old and reaches its highest point in school-age girls [17].

Accordingly, the aim of this study was to investigate whether *E. coli* strains responsible for cystitis in teenage girls (10–16 years old) with normal urinary tract (NUT) have different phylogenetic and phenotypic patterns from *E. coli* strains isolated from patients with cystitis and an abnormal urinary tract (AUT). The results demonstrated that *E. coli* strains derived from these two groups of patients have distinct genetic and phenotypic profiles. 

## 2. Results

### 2.1. Phylogenetic and Virulence Gene Profile

**Figure 1:**
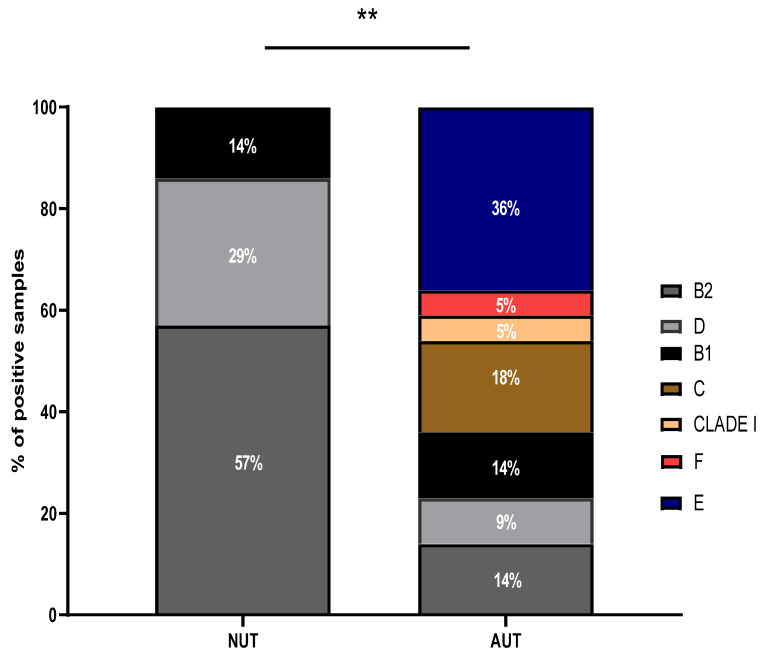
Distribution of phylogenetic groups among *E. coli* strains. The phylogenetic profile of *E. coli* strains isolated from the urine of teenage female patients with either normal or abnormal urinary tract was determined. NUT = Patients with normal urinary tract. AUT = Patients with abnormal urinary tract. ** Statistically significant (*p* ≤ 0.05) difference between experimental and control (strains isolated from patients with normal urinary tract) groups.

Phylogenetic analysis of the strains demonstrated that 57% of the isolates coming from patients with normal urinary tract (NUT) belonged to the phylo-group B2 and 29% to the phylo-group D (Figure 1, Table 1). In contrast, thirty six percent of the strains derived from patients with AUT belonged to the phylogroup E, 18% to the phylo-group C, 14% were B1 and 11% were characterized as F and CLADE I (Figure 1, Table 2). Only 23% of the strains derived from patients with AUT belonged to the phylogroups B2 and D. Strains isolated from the patients whose clinical picture developed to pyelonephritis were classified as B2 or D (Table 3).

**Figure 2:**
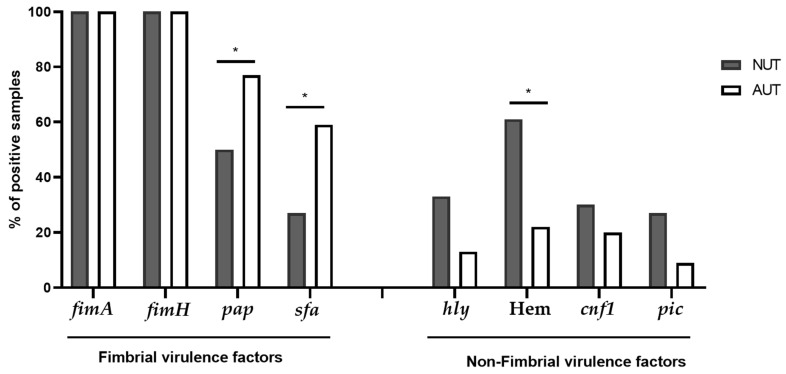
Distribution of virulence factors among *E. coli* strains. The presence of the virulence genes *fimA*, *fimH*, *pap, sfa*, *hly*, *cnf1*, *pic*, and were determined by PCR technique in *E. coli* strains isolated from the urine of teenage female patients with either normal or abnormal urinary tract. The ability of these strains to produce hemolysin was determined by the formation of a halo of lysed erythrocytes around the bacterial growth. NUT = Patients with normal urinary tract. AUT = Patients with abnormal urinary tract. Hem = hemolysin production. * Statistically significant (*p* ≤ 0.05) difference between experimental and control (strains isolated from patients with normal urinary tract) groups.

All isolates were positive for *fimH* and *fimA*. Seventy seven percent of the strains derived from patients with AUT were positive for *pap*, however, its prevalence decreased to 42% in strains derived from patients with NUT (Figure 2, Table 1 and Table 2). The same was observed for *sfa*, whose prevalence was 59% in strains derived from patients with AUT but decreased to 21% in strains isolated from patients with NUT (Figure 2, Table 1 and Table 2). The prevalence of hemolysin was 57% in the isolates derived from patients with NUT, and 22% in the strains derived from patients with AUT. The prevalence of *cnf*-1 and *pic* genes was low in all groups of patients (Figure 2, Table 1 and Table 2).

The strain O16:H5 (derived from a patient with NUT), OR:H18 (derived from a patient with AUT) and the strain ONT:H31 (derived from a patient with pyelonephritis), carried *aaiG* and *aaiA* genes, whereas the strains O16:H6, ONT:H18 and O20:H9 derived from patients with abnormal urinary tract carried *aggR* genes (Table 1, Table 2 and Table 3).

Three out of four patients with pyelonephritis were positive for the *papG* gene and hemolysin, whereas two out of four were positive to *sfa* gene (Table 3). 

### 2.2. Serogroups

**Figure 3:**
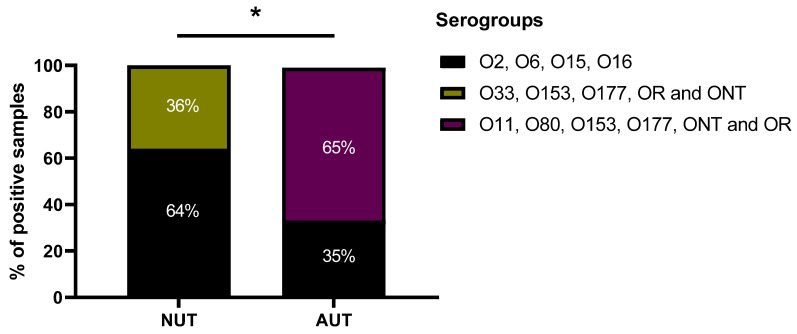
Serogroup determination of the *E. coli* strains. The serogroups of *E. coli* strains isolated from the urine of teenage female patients with either normal or abnormal urinary tract were determined. NUT = Patients with normal urinary tract. AUT = Patients with abnormal urinary tract. * Statistically significant (*p* ≤ 0.05) difference between experimental and control (strains isolated from patients with normal urinary tract) groups.

Sixty four percent of the isolates derived from patients with NUT belonged to the serogroups O2, O6, O15 and O16, whereas only 32% of the strains derived from patients with AUT belonged to these serogroups (Figure 3, Table 1).

Thirty percent of all isolates were either ONT (O non-typable) or OR (O rough strains). The prevalence of the serogroups 033, O153 and O177, OR and ONT was 35% in the strains derived from patients with NUT, while 64% of the isolates derived from patients with AUT belong to the serogroups O11, O80, O153, O177, ONT and OR (Figure 3).

### 2.3. Biofilm Formation on Abiotic Surfaces and Cell Adherence

**Figure 4:**
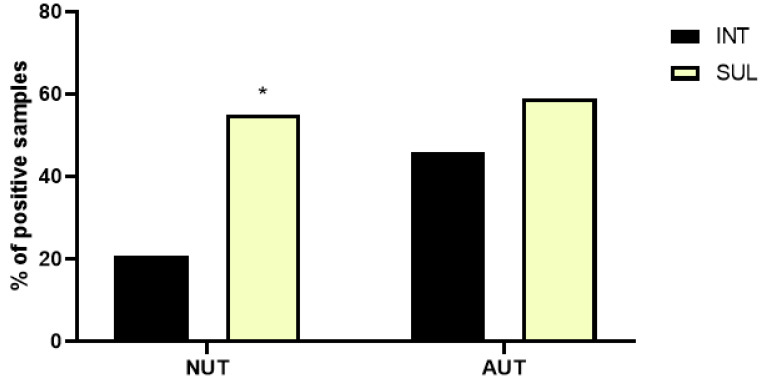
Determination of sulfonamide resistance and the presence of int1 in *E. coli* strains. The resistance to sulfonamide and the presence of *int1* gene were determined in *E. coli* strains isolated from the urine of teenage female patients with either normal or abnormal urinary tract. NUT = Patients with normal urinary tract. AUT = Patients with abnormal urinary tract. SUL = sulfonamide. * Statistically significant (*p* ≤ 0.05) difference in the same experimental group (strains isolated from patients with normal urinary tract).

The ability of the isolates derived from patients with NUT to form biofilm on PVC and polysterene was 50% and 35% respectively (Table 1). This ability increased to 90% (PVC) and 86% (polysterene) in strains derived from patients with AUT (Figure 4, Table 2). Eighty two percent of the strains isolated from patients with AUT were able to adhere to Vero cells, this percentage decreased to 43% in strains derived from patients with NUT (Table 2). All the strains isolated from the patients with pyelonephritis were able to form biofilm on polystyrene and PVC and adhere to epithelial cells (Table 3). 

### 2.4. Antimicrobial Profile—Presence of Int I

Antimicrobial analysis of the strains demonstrated that 50% of the isolates derived from patients with NUT were resistant to sulfonamide whereas 16% of theses samples were positive for the presence of *int1* (Figure 4, Table 4).

Fifty nine percent of the samples isolated from patients with AUT were resistant to sulfonamide and 45% of these isolates tested were positive for the presence of *int1* (Table 5).

The presence of *int-1* in the strains was only correlated to sulfonamide resistance in the isolates derived from patients with AUT (Figure 4, Table 5). Twenty percent of all isolates were resistant to amikacin, 15% were resistant to CIP, 10% were resistant to NAL and 27.5% were sensitive to all antibiotics tested (Table 4 and Table 5). 

Out of three isolates derived from patients with pyelonephritis were resistant to at least one of the antibiotics tested (Table 6).

## 3. Discussion

It has been demonstrated that besides ExPEC, pathogenic intestinal *E. coli* are also able to induce urinary tract intections (11, 12, 18). However, their association with the clinical picture of the patient is still unclear, especially in women, whose distance that separates the rectum from the urethra is very short, what makes them more exposed to infection by pathogenic intestinal microorganisms [18,19,20,21]. 

Although ExPEC are still responsible for most cases of UTI, the number of UTI caused by pathogenic intestinal *E. coli* has been increasing, but, the mechanism they use to induce infection in the urinary tract has not been elucidated. 

In the present work, however, a correlation between abnormal urinary tract and pathogenic intestinal *E. coli* was found in female patients with cystitis. For instance, *E. coli* strains isolated from the urine of patients with abnormal urinary tract (AUT) were associated with the phylo-groups E, C, F and CLAD I which are related to pathogenic intestinal *E. coli* [4,10,11,12]. In contrast, the *E. coli* strains isolated from patients with normal urinary tract (NUT) were associated with the phylogroups B2 and D which are related to virulent ExPEC [10]. 

It was also observed that a higher number of strains able to produce hemolysis were rather isolated from patients with NUT. It is likely that, in the case of patients with NUT, the hemolytic activity of UPEC helped to overcome physical, chemical and immune barriers in the host, which are weakened in patients with abnormal urinary tract. Probably, the hemolytic activity of UPEC, has also helped the isolates to ascend to the kidneys and there develop pyelonephritis, a phenomenon that has been observed in other studies [21].

In addition, *E. coli* isolated from the urine of patients with NUT were related to serogroups associated with virulent ExPEC such as O2, O6, O15 and O16 [13,14] whereas *E. coli* isolated from patients with abnormal urinary tract (AUT) were highly adherent strains whose serotypes have been described among emergent diarrheagenic *E. coli* strains [22,23,24,25,26].

Conversely, studies have shown that UPEC strains can carry genes attributed to diarrheagenic *E. coli*. This is well illustrated by the work of Abe and co-workers [14] who demonstrated that UPEC strains can encode *aggR* genes that are characteristic of typical-EAEC (enteroaggregative *E. coli*). In the present work, UPEC strains presented *aaiG*, *aaiA* and *aggR* genes that are preferentially associated with EAEC. 

In short, these data and several other studies suggest that the intestinal microbiota is a place where *E. coli* strains continuously exchange virulence factors between themselves, and by so doing, potentiates the emergence of intestinal hybrid pathogenic *E. coli* able to cause urinary tract infection [27,28,29,30].

The probability of intestinal pathogenic *E. coli* to cause urinary tract infection in patients with AUT is even greater in cases of dysbiosis where a direct link with the gut microbiota and the urethra is made [31]. In addition, the physical disabilities associated with these patients, such as urine retention and the necessity to use catheters make them more vulnerable to isolates able to form biofilm on these devices. The results demonstrated that more than 86% of the samples isolated from patients with AUT were able to produced biofilm on PVC and polystyrene, which are material used to produce catheters [16]. This is a very serious issue, since it has been demonstrated that the relapse of UTI in female patients aged >18 years old is associated with the ability of UPEC strains to form biofilm on these devices [32]. This situation can be aggravated by the fact that most of the strains isolated from patients with AUT were able to adhere to Vero cells, suggesting that they also have the potential to adhere to the kidneys. 

This scenario becomes even more critical taking into consideration the fact that several isolates were resistant to sulfonamide, amikacin and nalidixic acid, which are first choice classes of antibiotics to treat cystitis in women and children [33,34,35]. 

It is worth noting, that in the case of the isolates derived from patients with AUT, the correlation between the presence of integron I and sufonamide resistance is very important in terms of clinical surveillance alert, since integrons are gene cassettes that favors the emergence of antimicrobial resistance in pathogens [36].

Finally, the results obtained with male patients with normal and abnormal urinary tract, were not significantly different from one another (see Supplementary Materials). These results suggest that the proximity of the urethra with the rectum in female pediatric patients with abnormal urinary tract makes them more vulnerable to intestinal uropathogens. 

## 4. Material and Methods

### 4.1. Uropathogenic Escherichia coli—(UPEC) Strains

Seventy seven *E. coli* strains were isolated from the urine of female and male pediatric patients admitted to the “Hospital Infantil Darcy Vargas”, São Paulo, Brazil with a clinical profile of urinary tract infection. The age of the female patients ranged from 10 to 16 years old and the age of the male patients ranged from 0 to 4 years old. The study was reviewed and approved by the Ethics Committee of the Hospital Infantil Darcy Vargas (Certificate for Ethical Appreciation, CAAE 0005.0.350.3500-08) according to the 196/96 resolution and complementary resolutions (CONEP/CNS/MS). The strains were first identified by their biochemical profile in modified Rugai medium [37] as part of routine hospital procedures, and their identity subsequently confirmed by use of the API20E test (Biomerieux, France) at two different sites, the Instituto Adolfo Lutz, São Paulo Brazil and the Laboratory of Bacteriology of Instituto Butantan, São Paulo, Brazil. 

### 4.2. Clinical Picture of the Patients 

The clinical picture of the patients was classified as either cystitis of normal urinary tract (NUT), cystitis of abnormal urinary tract (AUT) or pyelonephritis. Patients with NUT were considered those with normal physiological and anatomical urinary tracts with infection of the lower urinary tract (bladder). Patients with AUT were considered those with either neurogenic bladder or any type of mal-formation or dysfunction of the urinary tract with infection of the lower urinary tract (bladder). Patients with pyelonephritis were considered those whose clinical picture had developed to kidney infection. 

### 4.3. Serotyping

The identification of somatic (O) and flagellar (H) antigens was performed by standard agglutination techniques, using specific antisera against O1 to O181 serogroups and against H1 to H56 [38]. The antisera utilized in this study were prepared in the Instituto Adolfo Lutz (São Paulo, Brazil) using reference *E. coli* strains. Cross reacting antisera were absorbed to prevent nonspecific agglutination.

### 4.4. Determination of E. coli Phylogenetic Groups 

The phylogenetic groups of the *E. coli* isolates were determined by a PCR-based technique using a combination of four DNA gene markers *chuA*, *yjaA*, *arpA* and the DNA fragment tspE4.C2 [10]. After amplification, the gene markers were analyzed by electrophoresis on agarose gel (0.7%) and visualized as DNA bands of 279, 211, 400 and 152-bp respectively. The phylogroups were determined based on the presence or absence of bands according to the criteria defined by Clermont et al., (2013) [10]. Accordingly, the isolates were classified as belonging to the phylogenetic groups A, B1, B2, C, D, E, F or CLADE-I. A 100 bp ladder was used as DNA molecular size pattern. 

### 4.5. PCR Amplification of Virulence Genes 

Genomic DNA was extracted from each *E. coli* isolate using DNA extraction and purification kits (Qiagen–DNA mini kit) from Qiagen Inc., California, CA, USA. Reactions were performed using the GeneAmp^®^ PCR System 9700 (Applied Biosystems, Foster City, CA, USA). The genes *pic*, *hly*, *fimA*, *fimH*, *pap*, *sfa*, *cnf1* and *int1* were PCR amplified using the primers and the conditions described in Table 7. The amplified markers were analyzed by electrophoresis on 0.8% agarose gel (GE Healthcare, Trasadingen, Switzerland) and visualized as amplicons of 1175, 596, 161, 508, 328, 410, 498 and 483 respectively.

The genes *pic*, *hly*, *fimA*, *fimH*, *pap*, *sfa*, *cnf1* and *int1* are responsible for coding of the following factors Pic, Hemolysin A, type I fimbria (adherence site), type 1 fimbria (conservative site), Pap, S fimbria, CNF-1 virulence factor and Integrase I.

The presence of *aggR*, *aaiG, aatA* and *aaiA* genes was determined as described by Andrade et al. (2014) [39].

### 4.6. Antimicrobial Resistance Profile

The antimicrobial susceptibility of the *E. coli* isolates was determined by the standard disk diffusion method [46], utilizing commercially available sensitivity discs and Mueller-Hinton Agar. The results were evaluated according to the CCLS-M100-S27, 2017 guidelines [47].

The following antibiotics were tested: amikacin (AMI), amoxicillin/clavulanic acid (AMC), ceftazidime (CAZ), ciprofloxacin (CIP), trimethoprim/sulfamethoxazole (SUT), aztreonam (ATM), imipenem (IPM), cefepima (CPM), cefotaxime (CTX), meropenem (MER), fosfomicin (FOS), gentamicin (GEN), nalidixic acid (NAL), ertapenem (ERT). For quality control the test was run against the following ATCC strains: *Escherichia coli* 25922 and *Pseudomonas aeruginosa* 27853.

### 4.7. Bacterial Adhesion to Epithelial Cells

The bacterial adhesion test was performed utilizing Vero cell line (cell line derived from the epithelial cells of the kidney of green monkey). The cell line was obtained from the Instituto Adolfo Lutz, São Paulo, Brazil, which was previously acquired from the American Type Culture Collection (CCL 2). For maintenance, the cells were grown in Dulbecco’s Modified Eagle Mediem (DMEM) supplemented with 10% calf serum, 1 mM L-glutamine, and 50 IU/mL penicillin-streptomycin. Briefly, the cells were grown in DMEM to 70% confluence on circular glass coverslips in 24-well tissue culture plates. Subsequently, 40 µL of *E. coli* culture (10^5^/mL) previously grown in Tryptic Soy Broth (TSB) for 18 h at 37 °C were added to the plates and incubated for 3 h. After incubation, the monolayers were washed six times with sterile PBS and then fixed with 100% methanol for 10 min, stained for 5 min with May-Grunwald stain (Merck) diluted 1:2 in Sorensen buffer, and finally stained for 20 min with Giemsa stain (Merck) diluted 1:3 in Sorensen buffer. The excess stain was discarded, and the coverslips with the stained cells were affixed to microscope slides for visualization by light microscopy (eyepiece, ×10; objective, ×100). This experiment was repeated twice with similar results.

### 4.8. Biofilm Formation on Abiotic Surfaces

The methodology utilized to determine the ability of the *E. coli* isolates to produce biofilm on abiotic surfaces was an adaptation of the methodology described by Sheikh et al. (2001) [48]. The experiment was performed in plates of polystyrene and PVC plates (Corning^®^, New York, NY, USA). Briefly, 190 µL of tryptic soy broth (TSB) were dispensed into each well of the plates and 10 µL of *E. coli* culture previously grown in LB (Luria Bertani) broth for 18 h at 37 °C were added in triplicate to the wells. After 24 h incubation, the plates were washed four times with PBS and the bacteria adhering to the plates were then fixed with 200 µL of 75% ethanol for 10 min. After incubation with ethanol, the plates were washed three times with PBS and the bacteria were stained with 0.5% crystal violet (CV) for 5 min, washed with PBS and then air-dried. The CV incorporated in the bacterial cells was solubilized by the addition of 95% ethanol (200 µL/well). After 2 min incubation at room temperature, 150 µL of the supernatant from each well were transferred to a microtiter plate and the extent of biofilm formation was determined by measuring the optical density at 595 nm in a Multiskan Ex type 355 (LabSystems, Vantaa, Finland). The UPEC prototype strain J96 was used as a positive control for biofilm formation. The strains were considered biofilm producers, when the absorbance was higher than 0.5. This experiment was repeated twice with similar results.

### 4.9. Identification of Hemolysin-Producing E. coli Strains 

Blood-agar culture plates were prepared according to Beutin (1991) [49]. Briefly, 1.5 g of TSA (Tryptic Soy Agar) re-suspended in a 10 mM solution of CaCl_2_ was autoclaved. When the temperature of the agar fell to 45 °C, sheep red cells previously washed three times in PBS pH 7.2 were then added to the agar to give a final concentration of 5%. The agar was added to Petri dish plates (20 mL per plate), left to solidify and kept at 4 °C until use. 

Subsequently, forty microliters of bacterial culture previously grown in TSB for 18 h at 37 °C were added to 3 mL of TSB and incubated overnight at 37 °C. After incubation, 100 µL of each bacterial culture were added in triplicate to the blood agar plates in aliquots of 10 µL each. The plates were then incubated for 18 h at 37 °C and the presence of hemolysin was determined by the formation of a halo of lysed erythrocytes around the bacterial growth. This experiment was repeated twice with similar results.

### 4.10. Statistic Analysis

Statistical analysis of the results (Figure 1, Figure 2, Figure 3 and Figure 4) was performed applying the unpaired two-tailed Student t test using Excel program software. The group of strains isolated from the urine of teenage female patients with cystitis and normal urinary tract (NUT) was used as a control. Results with *p* values lower than 0.05 (<0.05) were considered significant.

## 5. Conclusions

In summary, the results obtained in this study suggest that the acquisition of fimbrial adhesin genes, along with the capacity to adhere to epithelial cells, produce biofilm and resist to antibiotic therapy, allowed different intestinal pathogenic *E. coli* to successfully cause cystitis in teenage female patients with abnormal urinary tract. Conversely, the development of cystitis in female patients with normal urinary tract is induced mainly by classic extra intestinal virulent UPEC strains that have the potential to overcome the functional barriers imposed by their urinary system.

## Figures and Tables

**Table 1:** Serotype, phylogroup and virulence profile of *E. coli* strains derived from female patients with NUT.

Female Patients with NUT														
N°	Serotype	Phylo-Group	EAEC Genes	Virulence Factors										
				*fimA*	*fimH*	*pap*	*sfa*	*cnf1*	*pic*	*hly*	Hem	Biofilm		Adhesion
												PLT	PVC	
1	O33H:28	B1		+	+	-	-	-	+	+	+	-	-	-
2	O2:H4	D		+	+	+	-	-	-	+	+	+	+	-
3	O177:H21	B1		+	+	-	-	-	-	-	-	+	-	+
4	O6:H1	B2		+	+	+	+	+	-	-	+	+	+	-
5	O2:H6	B2		+	+	-	+	+	-	+	+	+	-	+
6	O16:H5	B2		+	+	-	-	nd	-	-	+	-	-	+
7	O2:H-	B2		+	+	+	-	+	+	+	+	-	-	-
8	ONT:HNT	B2		+	+	+	+	nd	-	-	+	-	-	-
9	O6:H-	B2		+	+	-	-	-	-	-	+	+	+	-
10	O16:H6	B2		+	+	+	-	-	-	-	-	-	-	-
11	O16:H5	B2	*aaiG*, *aaiA*	+	+	+	-	-	-	-	-	+	+	+
12	O153:H2	D		+	+	-	-	-	-	-	-	+	+	-
13	O15:H1	D		+	+	-	-	-	+	-	-	-	-	+
14	OR:H2	D		+	+	-	-	nd	-	-	-	-	-	+

O:H (serotype); *fimA* (operon encoding for type 1 fimbriae—adhesion site); *fimH* (operon encoding for type 1 fimbriae, constitutive part); *pap* (operon encoding for pili associated with pyelonephritis—P fimbriae), *sfa* (S fimbria); *cnf1* (operon encoding for cytotoxic necrotizing factor type I—CNF-1); *pic* (factor PIC); *hly* (operon encoding for hemolysin), Hem (hemolysin), biofilm production in polystyrene (PLT), polyvinyl chloride (PVC), adherent (+), not adherent (-), not done (nd). NUT: normal urinary tract.

**Table 2:** Serotypes, phylogroup and virulence profile of *E. coli* strains derived from female patients with AUT.

Female Patients with AUT														
N°	Serotype	Phylo-Group	EAEC Genes	Virulence Factors										
				*fimA*	*fimH*	*pap*	*sfa*	*cnf1*	*pic*	*hly*	Hem	Biofilm		Adhesion
												PLT	PVC	
1	ONT:HNT	C		+	+	+	+	nd	-	-	-	+	+	+
2	ONT:H18	E	*aggR*	+	+	-	+	-	-	-	-	+	+	+
3	O20:H9	CLADE I	*aggR*	+	+	-	+	-	-	-	-	+	+	+
4	O80:H26	C		+	+	+	-	-	-	-	-	+	+	+
5	O177:H21	B1		+	+	+	-		-	-	+	+	+	+
6	O2:H6	B2		+	+	+	+	nd	-	+	+	+	+	+
7	O2:H1	B2		+	+	+	+	nd	+	+	-	+	-	+
8	O6:H-	E		+	+	+	+	+	-	-	+	-	-	-
9	O16:H6	E	*aggR*	+	+	+	+	+	-	-	-	+	+	+
10	O6:H31	B2		+	+	+	+	nd	-	+	+	-	-	-
11	O16:H5	E		+	+	+	+	-	-	-	-	+	+	+
12	ONH:H-	C		+	+	+	-	nd	+	-	-	+	+	+
13	O2:H1	C		+	+	+	-	+	-	-	-	+	+	-
14	ONT:HNT	E		+	+	-	+	-	-	-	-	+	+	+
15	O11:H18	E		+	+	-	-	-	-	-	-	+	+	+
16	OR:H18	E	*aaiG*, *aaiA*	+	+	+	+	-	-	-	-	+	+	+
17	O86:H18	D		+	+	+	+	nd	-	-	-	+	+	-
18	OR:H18	D		+	+	+	+	nd	-	-	+	+	+	
19	O153:H10	F		+	+	+	-	-	-	-	-	+	+	+
20	O153:H18	E		+	+	+	-	-	-	-	-	+	+	+
21	ONT:H18	B1		+	+	+	-	-	-	-	-	+	+	+
22	ONT:H18	B1		+	+	-	-	nd	-	-	-	+	+	+

O:H (serotype); *fimA* (operon encoding for type I fimbriae—adhesion site); *fimH* (operon encoding for type 1 fimbriae, constitutive part); *pap* (operon encoding for pili associated with pyelonephritis—P fimbriae), *sfa* (S fimbria); *cnf1* (operon encoding for cytotoxic necrotizing factor type I—CNF-1); *pic* (factor Pic); *hly* (operon encoding for hemolysin), Hem (hemolysin), biofilm production in polystyrene (PLT) or in polyninyl chloride (PVC), adherent (+), not adherent (-), not done (nd). AUT: abnormal urinary tract.

**Table 3:** Serotype, phylogroup and virulence profile of *E. coli* strains derived from female patients with pyelonephritis.

Female Patients with Pyelonephritis														
N°	Serotype	Phylo-Group	EAEC Genes	Virulence Factors										
				*fimA*	*fimH*	*pap*	*sfa*	*cnf1*	*pic*	*hly*	Hem	Biofilm		Adhesion
												PLT	PVC	
1	ONT:H31	B2	*aaiG*, *aaiA*	+	+	+	+	-	-	+	+	+	+	-
2	OR:H18	D		+	+	+	-	nd	-	+	+	+	+	+
3	O6:H-	B2		+	+	+	+	-	-	+	+	+	+	-
4	O80:H26	B2		+	+	+	-	-	-	-	-	+	+	+

O:H (serotype); *fimA* (operon encoding for type I fimbriae—adhesion site); *fimH* (operon encoding for type 1 fimbriae, constitutive part); *pap* (operon encoding for pili associated with pyelonephritis—P fimbriae), *sfa* (S fimbria); *cnf1* (operon encoding for cytotoxic necrotizing factor type I—CNF-1); *pic* (factor Pic); *hly* (operon encoding for hemolysin), Hem (hemolysin), biofilm production in polystyrene (PLT) or in polyninyl chloride (PVC), adherent (+), not adherent (-), not done (nd).

**Table 4:** Antibiotic resistance profile of *E. coli* strains derived from female patients with NUT.

Antibiotic Resistance-Female Patients with NUT																	
N°	Serotype	*int1*	SUT	ATM	CAZ	CIP	IPM	AMC	CTX	CPM	MER	FOS	GEN	AMI	NAL	ERT	Total Antibiotic Resistance
1	O33H:28	-	R	S	S	S	S	S	S	S	S	S	S	S	S	S	SUT
2	O2:H4	+	R	S	S	S	S	S	S	S	S	S	S	S	S	S	SUT
3	O177:H21	+	S	S	S	R	S	S	S	S	S	S	S	S	R	S	CIP, NAL
4	O6:H1	-	R	S	S	S	S	S	S	S	S	S	S	S	S	S	SUT
5	O2:H6	-	S	S	S	S	S	S	S	S	S	S	S	S	S	S	S
6	O16:H5	-	R	S	S	S	S	R	S	S	S	S	S	S	S	S	SUT, AMC
7	O2:H-	-	R	S	S	S	S	S	S	S	S	S	S	S	S	S	SUT
8	ONT:HNT	nd	R	S	S	R	S	nd	S	S	S	nd	R	S	nd	nd	SUT, CIP, GEN
9	O6:H-	-	S	S	S	S	S	S	S	S	S	S	S	S	S	S	S
10	O16:H6	-	R	S	S	S	S	S	S	S	S	S	S	S	S	S	SUT
11	O16:H5	-	S	S	S	S	S	S	S	S	S	S	S	S	S	S	S
12	O153:H2	-	S	S	S	S	S	S	S	S	S	S	S	S	S	S	S
13	O15:H1	-	S	S	S	S	S	nd	S	S	S	nd	S	S	nd	nd	S
14	OR:H2	nd	S	R	R	R	S	nd	S	R	S	nd	R	R	nd	nd	ATM, CAZ, CIP, CPM, GEN, AMIC

The following antibiotics were tested: trimethoprim/sulfamethoxazole (SUT), aztreonam (ATM), ceftazidime (CAZ), ciprofloxacin (CIP), imipenem (IPM), amoxicillin/clavulanic acid (AMC), cefotaxime (CTX), cefepime (CPM), meropenem (MER), fosfomicin (FOS), gentamicin (GEN), amikacin (AMI), nalidixic acid (NAL), ertapenem (ERT), not done (nd), resistant (R), sensitive (S). *int1* (operon encoding for integrase 1). For quality control the test was run against the following ATCC strains: *Escherichia coli* 25922 and *Pseudomonas aeruginosa* 27853. NUT: Patients with normal urinary tract.

**Table 5:** Antibiotic resistance profile of *E. coli* strains derived from patients with AUT.

Antibiotic Resistance–Female Patients with AUT																	
N°	Serotype	*int1*	SUT	ATM	CAZ	CIP	IPM	AMC	CTX	CPM	MER	FOS	GEN	AMI	NAL	ERT	Total Antibiotic Resistance
1	ONT:HNT	-	S	S	S	S	S	S	S	S	S	S	S	S	S	S	S
2	ONT:H18	+	R	S	S	S	S	S	S	S	S	S	S	S	S	S	SUT
3	O20:H9	+	R	S	S	R	S	S	S	S	S	S	S	S	R	S	SUT, CIP, NAL
4	O80:H26	-	R	S	S	S	S	S	S	S	S	S	S	S	S	S	SUT
5	O177:H21	-	S	S	S	S	S	S	S	S	S	S	S	S	R	S	NAL
6	O2:H6	nd	S	R	R	R	R	nd	S	R	S	nd	R	R	nd	nd	ATM, CAZ, CIP, IPM, CPM, GEN, AMI
7	O2:H1	nd	R	S	S	S	S	nd	S	S	S	nd	S	S	nd	nd	SUT
8	O6:H-	-	S	S	S	S	S	S	S	S	S	S	S	S	S	S	S
9	O16:H6	+	R	S	S	S	S	S	S	S	S	S	S	S	S	S	SUT
10	O6:H31	nd	S	R	R	R	S	nd	S	S	S	nd	S	S	nd	nd	ATM, CAZ, CIP
11	O16:H5	+	R	S	S	S	S	S	S	S	S	S	S	S	S	S	SUT
12	ONH:H-	nd	S	S	S	S	S	nd	S	S	S	nd	S	R	nd	nd	S
13	O2:H1	-	R	S	S	S	S	S	S	S	S	S	S	S	S	S	SUT
14	ONT:HNT	-	S	S	S	S	S	S	S	S	S	S	S	S	S	S	S
15	O11:H18	+	R	S	S	S	S	R	S	S	S	S	S	S	S	S	SUT, AMC
16	OR:H18	+	R	S	S	S	S	R	S	S	S	S	S	S	S	S	SUT, AMC
17	O86:H18	nd	S	S	S	S	S	nd	S	S	S	nd	S	S	nd	nd	S
18	OR:H18	nd	R	S	S	S	R	nd	R	S	R	nd	S	R	nd	nd	SUT, IPM, CTX, MER, AMI
19	O153:H10	-	R	S	S	S	S	R	S	S	S	S	S	S	S	S	SUT
20	O153:H18	-	R	S	S	S	S	S	S	S	S	S	S	S	S	S	SUT
21	ONT:H18	+	R	S	S	S	S	S	S	S	S	S	S	S	R	S	SUT, NAL
22	ONT:H18	nd	S	S	S	S	S	nd	S	S	S	nd	S	S	nd	nd	S

The following antibiotics were tested: trimethoprim/sulfamethoxazole (SUT), aztreonam (ATM), ceftazidime (CAZ), ciprofloxacin (CIP), imipenem (IPM), amoxicillin/clavulanic acid (AMC), cefotaxime (CTX), cefepime (CPM), meropenem (MER), fosfomicin (FOS), gentamicin (GEN), amikacin (AMI), nalidixic acid (NAL), ertapenem (ERT), not done (nd), resistant (R), sensitive (S). *int1* (operon encoding for integrase 1). For quality control the test was run against the following ATCC strains: *Escherichia coli* 25922 and *Pseudomonas aeruginosa* 27853. AUT: Patients with abnormal urinary tract.

**Table 6:** Antibiotic resistance profile of *E. coli* strains derived from female patients with pyelonephritis.

Antibiotic Resistance–Female Patients with Pyelonephritis																	
Nº	Serotype	*int1*	SUT	ATM	CAZ	CIP	IPM	AMC	CTX	CPM	MER	FOS	GEN	AMI	NAL	ERT	Total Antibiotic Resistance
1	ONT:H31	+	R	S	S	S	S	R	S	S	S	S	S	S	S	S	SUT, AMC
2	OR:H18	nd	R	S	S	S	R	nd	R	S	R	nd	S	R	nd	nd	SUT, IPM, CTX, MER, AMI
3	O6:H-	-	S	S	S	S	S	S	S	S	S	S	R	S	S	S	S
4	O80:H26	nd	S	S	S	S	S	nd	S	S	S	nd	S	S	nd	nd	S

The following antibiotics were tested: trimethoprim/sulfamethoxazole (SUT), aztreonam (ATM), ceftazidime (CAZ), ciprofloxacin (CIP), imipenem (IPM), amoxicillin/clavulanic acid (AMC), cefotaxime (CTX), cefepime (CPM), meropenem (MER), fosfomicin (FOS), gentamicin (GEN),amikacin (AMI), nalidixic acid (NAL), ertapenem (ERT), not done (nd), resistant (R), sensitive (S). *int1* (operon encoding for integrase 1). For quality control the test was run against the following ATCC strains: *Escherichia coli* 25922 and *Pseudomonas aeruginosa* 27853.

**Table 7:** Primers sequences of virulence genes, annealing temperatures, and size of PCR product.

Genes	Primer Sequence (5′- 3′)	Annealing Temperatures (°C)	Amplicon Size (bp)	References
*fim* *A*	CTGTCGGCTCTGTCCCTCAGT GATGCGGTACGAACCTGTCCTAA	65	161	[40]
*fim* *H*	TGCAGAACGGATAAGCCGTGG GCAGTCACCTGCCCTCCGGTA	63	508	[41]
*pap*	GACGGCTGTACTGCAGGGTGTGGCG ATATCCTTTCTGCAGGGATGCAATA	50	328	[42]
*sfa*	CTCCGGAGAACTGGGTGCATCTTAC CGGAGGAGTAATTACAAACCTGGCA	50	410	[42]
*cnf1*	AAGATGGAGTTTCCTATGCAGGAG CATTCAGAGTCCTGCCCTCATTATT	61	498	[43]
*int* *1*	ACATGCGTGTAAATCATCGTCG GGGTCAAGGATCTGGATTTCG	62	483	[44]
*pic*	GGGTATTGTCCGTTCCGAT ACAACGATACCGTCTCCCG	55	1175	[45]
*hly*	GGTGCAGCAGAAAAAGTTGTAG TCTCGCCTGATAGTGTTTGGTA	57	596	M10133(hlyA)

## Data Availability

The data presented in this study are available on request from the corresponding author. The data are not available in the repository of the Butantan Institute (https://repositorio.butantan.gov.br (accessed on 23 December 2021).

## Supplementary Materials

The following supporting information can be downloaded at: https://www.mdpi.com/article/10.3390/pathogens11020231/s1. Table S1: Serotype, phylogroup and virulence profile of *E. coli* strains derived from male patients with NUT. Table S2: Serotypes, phylogroup and virulence profile of *E. coli* strains derived from male patients with AUT. Table S3: Serotypes, phylogroup and virulence profile of *E. coli* strains derived from male patients with Pyelonepritis. Table S4: Antibiotic resistance profile of *E. coli* strains derived from male patients with NUT. Table S5: Antibiotic resistance profile of *E. coli* strains derived from male patients with AUT. Table S6: Serotypes, phylogroup and virulence profile of *E. coli* strains derived from male patients with Pyelonepritis.
