# Supplementary material for: Escherichia coli Strains Responsible for Cystitis in Female Pediatric Patients with Normal and Abnormal Urinary Tracts Have Different Virulence Profiles
Source: Pathogens. 2022 Feb 10;11(2):231. doi: 10.3390/pathogens11020231 (PMC8876236; doi:10.3390/pathogens11020231)
Supplement: Supplementary file 1 [file pathogens-11-00231-s001.zip › pathogens-1553512-supplementary.pdf]

Supplementary tables

Table S1. Serotype, phylogroup and virulence profile of *E. coli* strains derived from male patients with NUT.

MALE PATIENTS WITH NUT														
**Nº**	**SEROTYPE**	**PHYLO-GROUP**	**EAEC GENES**	**VIRULENCE FACTORS**										
				fimA	***fimH***	***pap***	***sfa***	***cnfI***	***pic***	***hly***	**Hem**	**Biofilm**		**Adhesion**
												**PLT**	**PVC**	
1	O1:H7	B2		+	+	+	-	-	-	-	-	+	+	+
2	ONT:HNT	B2		+	+	+	-	nd	-	+	+	+	+	-
3	O2:H6	B2		+	+	+	-	-	+	+	+	+	+	+
4	O6:H31	B2		+	+	-	-	+	-	-	-	-	-	-
5	D49	D		+	+	-	-	-	-	-	-	+	+	-
6	OR:H23	B2		+	+	-	-	-	-	-	-	+	+	+
7	OR:H-	B2		+	+	+	-	-	-	-	-	+	+	+
8	O2:H1	D		+	+	+	-	nd	-	-	-	+	+	-
9	ONT:H5	B2		+	+	+	+	nd	-	-	+	+	+	+
10	O86:H8	B1		+	+	-	-	-	-	-	-	+	+	+

O:H (serotype); *fimA* (operon encoding for type 1 fimbriae - adhesion site); *fimH* (operon encoding for type 1 fimbriae, constitutive part); *pap* (operon encoding for pili associated with pyelonephritis – P fimbriae), *sfa* (S fimbria); *cnf1* (operon encoding for cytotoxic necrotizing factor type I – CNF-1); *pic* (factor PIC); *hly* (operon encoding for hemolysin), Hem (hemolysin), biofilm production in polystyrene (PLT), polyvinyl chloride (PVC), adherent (+), not adherent (-), not done (nd). NUT: normal urinary tract.

Table S2. Serotypes, phylogroup and virulence profile of *E. coli* strains derived from male patients with AUT.

MALE PATIENTS WITH AUT														
**Nº**	**SEROTYPE**	**PHYLO-GROUP**	**EAEC GENES**	**VIRULENCE FACTORS**										
				fimA	***fimH***	***pap***	***sfa***	***cnf-1***	***pic***	***hly***	**Hem**	**Biofilm**		**Adhesion**
												PLT	PVC	
1	ONT:H33	A		+	+	-	-	-	-	-	-	+	+	-
2	O115:H10	B1		+	+	-	-	nd	-	-	-	+	+	-
3	O2:H6	B2		+	+	+	-	+	-	+	+	+	-	+
4	O6:H31	B2		+	+	+	-	+	-	+	+	+	+	-
5	O141:H-	B2		+	+	-	+	-	-	-	-	+	+	+
6	O2:H1	B2		+	+	-	-	+	-		-	-	-	-
7	O6:H31	B2	*aaiG*, *aatA*, *aaiA*, *aggR*,	+	+	+	+	+	-	+	+	+	+	-
8	O2:H1	B2		+	+	-	-	+	+	+	+	-	-	+
9	O2:H1	B2		+	+	-	-	+	+	-	-	+	+	-
10	O2:H-	B2	*aggR*	+	+	+	+	+		+	+	+	+	-
11	OR:H10	E		+	+	+	+	-	-	+	+	+	-	+
12	O18:H-	B2		+	+	+	+	-	-	+	+	-	+	+
13	OR:H1	B2		+	+	-	+	+	-	+	+	-	-	-
14	ONT:H2	A		+	+	-	-	-	-	-	-	+	+	+
15	O20:H9	B2		+	+	-	+	-	-	-	-	+	+	+
16	O2:H4	D		+	+	+	-	-	-	+	+	+	+	+
17	OR:H9	D		+	+	-	-	-	-	-	-	+	+	+
18	O6:H1	B2		+	+	+	-	+	-	+	+	+	+	-

Nº	SEROTYPE	PHYLO-GROUP	EAEC GENES	fimA	fimH	pap	sfa	cnf1	pic	hly	Hem	Biofilm PLT	Biofilm PVC	Adhesion
19	O80:H26	B1		+	+	+	-	-	-	-	-	+	+	+
20	OR:H26	A		+	+	+	-	-	-	-	-	+	+	+
21	OR:H26	E		+	+	-	+	-	-	-	-	+	-	+
22	ONT:H18	D		+	+	+	+	nd	-	-	-	+	+	+

O:H (serotype); *fimA* (operon encoding for type 1 fimbriae - adhesion site); *fimH* (operon encoding for type 1 fimbriae, constitutive part); *pap* (operon encoding for pili associated with pyelonephritis – P fimbriae), *sfa* (S fimbria); *cnf1* (operon encoding for cytotoxic necrotizing factor type I – CNF-1); *pic* (factor PIC); *hly* (operon encoding for hemolysin), Hem (hemolysin), biofilm production in polystyrene (PLT), polyvinyl chloride (PVC), adherent (+), not adherent (-), not done (nd). AUT: Abnormal urinary tract.

Table S3. Serotypes, phylogroup and virulence profile of *E. coli* strains derived from male patients with Pyelonepritis.

MALE PATIENTS WITH PYELONEPHRITIS														
Nº	SEROTYPE	PHYLO-GROUP	EAEC GENES	VIRULENCE FACTORS										
				fimA	*fimH*	*pap*	*sfa*	*cnf1*	*pic*	*hly*	Hem	Biofilm		Adhesion
												PLT	PVC	
1	O18:H31	B2		+	+	-	-	-	-	-	+	-	+	+
2	O153:H31	E		+	+	-	-	nd	-	-	-	+	+	-
3	O34:HNT	B2		+	+	-	-	-	-	-	-	+	+	+
4	O6:H1	B2		+	+	+	-	nd	-	-	+	+	+	-
5	O7:H-	D		+	+	+	+	nd	-	-	-	+	+	+

O:H (serotype); *fimA* (operon encoding for type 1 fimbriae - adhesion site); *fimH* (operon encoding for type 1 fimbriae, constitutive part); *pap* (operon encoding for pili associated with pyelonephritis – P fimbriae), *sfa* (S fimbria); *cnf1* (operon encoding for cytotoxic necrotizing factor type I – CNF-1); *pic* (factor PIC); *hly* (operon encoding for hemolysin), Hem (hemolysin), biofilm production in polystyrene (PLT), polyvinyl chloride (PVC), adherent (+), not adherent (-), not done (nd).

Table S4. Antibiotic resistance profile of *E. coli* strains derived from male patients with NUT.

ANTIBIOTIC RESISTANCE – MALE PATIENTS WITH NUT																
Nº	SEROTYPE	*int1*	SUT	ATM	CAZ	CIP	IPM	AMC	CTX	CPM	MER	FOS	GEN	AMI	NAL	ERT	Total antibiotic resistance
1	O1:H7	-	S	S	S	S	S	S	S	S	S	S	S	S	S	S	S
2	ONT:HNT	-	S	S	S	S	S	S	S	S	S	S	S	S	S	S	S
3	O2:H6	-	R	S	S	S	S	S	S	S	S	S	S	S	S	S	SUL
4	O6:H31	+	R	S	S	S	S	R	S	S	S	S	S	S	S	S	SUL
5	nd	-	S	S	S	S	R	S	S	S	S	S	S	S	R	S	SUL, NAL
6	OR:H23	+	nd	nd	nd	nd	nd	nd	nd	nd	nd	nd	nd	nd	nd	nd	nd
7	OR:H-	-	S	S	S	S	S	S	S	S	S	S	S	S	S	S	S
8	O2:H1	nd	nd	nd	nd	nd	nd	nd	nd	nd	nd	nd	nd	nd	nd	nd	nd
9	ONT:H15	nd	nd	nd	nd	nd	nd	nd	nd	nd	nd	nd	nd	nd	nd	nd	nd
10	O86:H8	+	R	S	S	R	S	S	S	S	S	S	S	S	R	S	SUL, CIP. NAL

The following antibiotics were tested: trimethoprim/sulfamethoxazole (SUT), aztreonam (ATM), ceftazidime (CAZ), ciprofloxacin (CIP), imipenem (IPM), amoxicillin/clavulanic acid (AMC), cefotaxime (CTX), cefepime (CPM), meropenem (MER), fosfomicin (FOS), gentamicin (GEN), amikacin (AMI), nalidixic acid (NAL), ertapenem (ERT), not done (nd), resistant (R), sensitive (S). *int1* (operon encoding for integrase 1). For quality control the test was run against the following ATCC strains: *Escherichia coli* 25922 and *Pseudomonas aeruginosa* 27853. NUT: Patients with normal urinary tract.

Table S5. – Antibiotic resistance profile of *E. coli* strains derived from male patients with AUT.

№	SEROTYPE	*int1*	SUT	ATM	CAZ	CIP	IPM	AMC	CTX	CPM	MER	FOS	GEN	AMI	NAL	ERT	Total antibiotic resistance
**ANTIBIOTIC RESISTANCE – MALE PATIENTS WITH AUT**																	
1	ONT:H33	-	S	S	S	S	S	S	S	S	S	S	S	S	S	S	S
2	O115:H10	nd	nd	nd	nd	nd	nd	nd	nd	nd	nd	nd	nd	nd	nd	nd	nd
3	O2:H6	-	S	S	S	S	S	R	S	S	S	S	S	S	S	S	AMC
4	O6:H31	-	R	S	S	S	S	R	S	S	S	S	S	S	S	S	SUL, AMC
5	O141:H-	+	nd	nd	nd	nd	nd	nd	nd	nd	nd	nd	nd	nd	nd	nd	nd
6	O2:H1	-	R	S	S	S	S	S	S	S	S	S	S	S	S	S	SUL
7	O6:H31	+	R	S	S	S	S	R	S	S	S	S	S	S	S	S	SUL, AMC
8	O2:H1	-	R	R	S	S	S	S	S	S	S	S	S	R	S	S	SUT, ATM.AMI
9	O2:H1	-	R	S	S	S	S	S	S	S	S	S	S	S	S	S	SUL
10	O2:H-	-	R	S	S	S	S	S	S	S	S	S	S	S	S	S	SUL
11	OR:H10	+	R	S	S	S	S	S	S	S	S	S	S	S	S	S	SUL
12	O18:H-	+	R	S	S	S	S	S	S	S	S	S	S	S	S	S	SUL
13	OR:H1	+	R	S	S	S	S	S	S	S	S	S	S	S	R	S	SUL, NAL
14	ONT:H2	-	S	S	S	S	S	S	R	S	S	S	S	S	S	S	CTX
15	O20:H9	-	R	S	S	R	S	S	S	S	S	S	S	S	R	S	SUL, CIP, NAL
16	O2:H4	-	R	S	S	S	S	S	S	S	S	S	S	S	S	S	SUL
17	OR:H9	-	S	S	S	S	S	S	S	S	S	S	S	S	S	S	nd
18	O6:H1	+	R	S	S	S	S	R	S	S	S	S	S	S	S	S	SUL, AMC
19	O80:H26	-	R	S	S	S	S	S	S	S	S	S	S	S	R	S	SUL, NAL
20	OR:H26	-	S	S	S	S	S	S	S	S	S	S	S	S	S	S	S
21	OR:H26	-	R	S	S	S	S	R	S	S	S	S	S	S	S	S	SUL, AMC
22	ONT:H18	nd	nd	nd	nd	nd	nd	nd	nd	nd	nd	nd	nd	nd	nd	nd	nd

The following antibiotics were tested: trimethoprim/sulfamethoxazole (SUT), aztreonam (ATM), ceftazidime (CAZ), ciprofloxacin (CIP), imipenem (IPM), amoxicillin/clavulanic acid (AMC), cefotaxime (CTX), cefepime (CPM), meropenem (MER), fosfomicin (FOS), gentamicin (GEN), amikacin (AMI), nalidixic acid (NAL), ertapenem (ERT), not done (nd), resistant (R), sensitive (S). *int1* (operon encoding for integrase 1). For quality control the test was run against the following ATCC strains: *Escherichia coli* 25922 and *Pseudomonas aeruginosa* 27853. AUT: Patients with abnormal urinary tract.

Table S6. – Serotypes, phylogroup and virulence profile of *E. coli* strains derived from male patients with Pyelonepritis.

№	SEROTYPE	*int1*	SUT	ATM	CAZ	CIP	IPM	AMC	CTX	CPM	MER	FOS	GEN	AMI	NAL	ERT	Total antibiotic resistance
**ANTIBIOTIC RESISTANCE – MALE PATIENTS WITH PYELONEPHRITIS**																	
1	O18:H31	+	R	S	S	S	S	S	S	S	S	S	S	S	R	S	SUL, NAL
2	O153:H31	nd	nd	nd	nd	nd	nd	nd	nd	nd	nd	nd	nd	nd	nd	nd	nd
3	O34:HNT	-	R	S	S	S	S	S	S	S	S	S	S	S	R	S	SUL, NAL
4	O6:H1	nd	nd	nd	nd	nd	nd	nd	nd	nd	nd	nd	nd	nd	nd	nd	nd
5	O7:H-	nd	nd	nd	nd	nd	nd	nd	nd	nd	nd	nd	nd	nd	nd	nd	nd

The following antibiotics were tested: trimethoprim/sulfamethoxazole (SUT), aztreonam (ATM), ceftazidime (CAZ), ciprofloxacin (CIP), imipenem (IPM), amoxicillin/clavulanic acid (AMC), cefotaxime (CTX), cefepime (CPM), meropenem (MER), fosfomicin (FOS), gentamicin (GEN), amikacin (AMI), nalidixic acid (NAL), ertapenem (ERT), not done (nd), resistant (R), sensitive (S). *int1* (operon encoding for integrase 1). For quality control the test was run against the following ATCC strains: *Escherichia coli* 25922 and *Pseudomonas aeruginosa* 27853.
